# Liquid Biopsies in Solid Cancers: Implementation in a Nordic Healthcare System

**DOI:** 10.3390/cancers13081861

**Published:** 2021-04-13

**Authors:** Oddmund Nordgård, Rakel Brendsdal Forthun, Morten Lapin, Bjørn Henning Grønberg, Karl Henning Kalland, Reidun Kristin Kopperud, Liv Cecilie Vestrheim Thomsen, Kjersti Tjensvoll, Bjørnar Gilje, Bjørn Tore Gjertsen, Randi Hovland

**Affiliations:** 1 Department of Hematology and Oncology, Stavanger University Hospital, 4011 Stavanger, Norway; morten.lapin@sus.no (M.L.); kjersti.tjensvoll@sus.no (K.T.); bjornar.gilje@sus.no (B.G.); 2 Department of Chemistry, Bioscience and Environmental Engineering, University of Stavanger, 4021 Stavanger, Norway; 3 Department of Medical Genetics, Haukeland University Hospital, 5021 Bergen, Norway; rakel.brendsdal.forthun@helse-bergen.no (R.B.F.); randi.hovland@helse-bergen.no (R.H.); 4 Section of Cancer Genomics, Haukeland University Hospital, 5021 Bergen, Norway; 5 Department of Clinical and Molecular Medicine, NTNU, Norwegian University of Science and Technology, 7491 Trondheim, Norway; bjorn.h.gronberg@ntnu.no; 6 Department of Oncology, St. Olav’s Hospital, Trondheim University Hospital, 7030 Trondheim, Norway; 7 Centre for Cancer Biomarkers CCBIO, Department of Clinical Science, University of Bergen, 5021 Bergen, Norway; Kalland@uib.no (K.H.K.); Reidun.Kristin.Kopperud@uib.no (R.K.K.); Liv.Vestrheim@uib.no (L.C.V.T.); bjorn.tore.gjertsen@helse-bergen.no (B.T.G.); 8 Department of Microbiology, Haukeland University Hospital, 5021 Bergen, Norway; 9 Department of Internal Medicine, Hematology Section, Haukeland University Hospital, 5021 Bergen, Norway

**Keywords:** liquid biopsies, cancer, circulating tumor DNA, circulating tumor cells

## Abstract

**Simple Summary:**

We here review liquid biopsy methods and their use in the diagnostics and treatment of patients with solid cancers. More specifically, circulating tumor DNA, circulating tumor cells, and their current and future clinical applications are considered. Important factors for further integration of liquid biopsy methods in clinical practice are discussed, with a special focus on a Nordic Healthcare system.

**Abstract:**

Liquid biopsies have emerged as a potential new diagnostic tool, providing detailed information relevant for characterization and treatment of solid cancers. We here present an overview of current evidence supporting the clinical relevance of liquid biopsy assessments. We also discuss the implementation of liquid biopsies in clinical studies and their current and future clinical role, with a special reference to the Nordic healthcare systems. Our considerations are restricted to the most established liquid biopsy specimens: circulating tumor DNA (ctDNA) and circulating tumor cells (CTC). Both ctDNA and CTCs have been used for prognostic stratification, treatment choices, and treatment monitoring in solid cancers. Several recent publications also support the role of ctDNA in early cancer detection. ctDNA seems to provide more robust clinically relevant information in general, whereas CTCs have the potential to answer more basic questions related to cancer biology and metastasis. Epidermal growth factor receptor-directed treatment of non-small-cell lung cancer represents a clinical setting where ctDNA already has entered the clinic. The role of liquid biopsies in treatment decisions, standardization of methods, diagnostic performance and the need for further research, as well as cost and regulatory issues were identified as factors that influence further integration in the clinic. In conclusion, substantial evidence supports the clinical utility of liquid biopsies in cancer diagnostics, but further research is still required for a more general application in clinical practice.

## 1. Introduction

Examination of body fluids to diagnose disease has accompanied medicine from its very beginning. The timeline stretches from Hippocrates around 300 B.C., who suggested tasting the patient’s urine [1], to the plethora of diagnostic tests available in a medical biochemistry laboratory today [2]. The more recent advent of the term liquid biopsy relates to the development of new technologies that enable access to tumor cells and the tumor genome through samples of body fluids [3], analyses which have traditionally been performed using tissue biopsies.

A liquid biopsy is in principle a sample of any body fluid that may contain genetic material from a tumor; for instance, blood, urine, feces, saliva or cerebrospinal fluid [4]. The entities typically searched for are intact tumor cells, cell-free nucleic acids and tumor-derived extracellular vesicles. The applications reach beyond tumor genome characterization and include almost all aspects of cancer diagnostics; detection, characterization, prognostic stratification, therapy choices and response monitoring [5]. Here, we review blood-based liquid biopsy techniques and give examples of clinical applications and challenges in solid cancers, with a special focus on implementation in clinical studies and current and future roles in the health services. Our considerations will be restricted to the most established liquid biopsy specimens: circulating tumor DNA (ctDNA) and circulating tumor cells (CTC).

## 2. Methodological Approaches to Liquid Biopsies

### 2.1. Detection and Characterization of ctDNA 

Tumor-derived DNA fragments present in the circulation of cancer patients have received much focus in recent clinical cancer research [6]. A major challenge in the detection of ctDNA is that it co-exists with wild-type cell-free DNA (cfDNA), predominantly shedded from hematopoietic cells. Whereas ctDNA can make up the majority of the total cfDNA in many late-stage cancers, it comprises only minute amounts of the total cfDNA in patients with early-stage cancers [7]. Thus, sensitive detection methods are necessary to distinguish ctDNA from background hematopoietic cell-derived cfDNA. Other challenges in ctDNA detection are the overall low concentration of cfDNA in plasma, its high degree of fragmentation, and the heterogeneity in cancer-specific mutations.

Tumor-associated aberrations such as point mutations [7,8,9], insertion/deletions [10,11], copy number variations (CNV) [8,9], translocations [10], and epigenetic changes such as methylations [11,12], have all been targeted in order to detect and characterize ctDNA (Table 1). The most commonly used method for ctDNA detection is to target point mutations in genes frequently mutated in cancer. This is usually performed using highly sensitive detection methods such as droplet digital PCR (ddPCR) and BEAMing, either by screening for hotspot mutations or by using a tumor tissue-informed approach [13,14,15]. For patients where the mutation profile is not known due to lack of tumor tissue, or where subclonal evolution or acquired resistance to treatment is investigated, simultaneous detection of multiple mutations by deep sequencing of large gene panels is a potent tool to detect and characterize ctDNA on a larger scale [16,17,18]. Early deep sequencing-based methods had low sensitivity due to PCR and sequencing errors and were not able to detect low fractions of ctDNA (<1%). The addition of unique molecular identifiers (UMI) to reduce these errors has largely defeated this issue. The sensitivity of deep sequencing is now comparable to ddPCR, which has long been considered the “gold standard” for ctDNA detection, albeit at a higher cost per sample and longer turnaround time [19,20]. A challenge with ctDNA sequencing is that the results can be confounded by somatic mutations in non-malignant hematopoietic cells, known as clonal hematopoiesis of indeterminate potential (CHIP), a phenomenon that increases with age [21,22]. It is therefore highly recommended to analyze matched cell-free and white blood cell DNA to remove CHIP variants, in addition to germline variants.

For patients without detectable point mutations and cancers prone to CNVs, it might be useful to investigate CNVs as markers for ctDNA detection, although the sensitivity for CNV detection is low compared to point mutations and insertions/deletions [9,23]. Furthermore, recent reports have suggested that targeting differentially methylated regions (DMRs) can improve the detection of ctDNA [11,12]. In contrast to somatic mutations, which are often limited in number, epigenetic changes such as DMRs are frequent and also tissue- and cancer type-specific [24]. The sensitivity can be further improved by increasing the number of DMRs analyzed, enabling detection of ctDNA even at low variant allele frequencies [12].

### 2.2. Detection of CTCs

CTCs are tumor cells that have left the primary tumor or metastatic sites and entered the bloodstream by intravasation [25]. CTCs are extremely rare compared to other cells in the blood of patients with cancer (110,000 per liter) [26], so extensive enrichment is required to detect them. Current CTC-enrichment methods (Table 2) are based on either physical properties (size, density, deformability, and electrical charge) or biological features (cell-surface protein expression, invasive capacity, and viability) [27]. The CellSearch system, which is based on biological features, remains the gold standard for enrichment and detection of CTCs. Paramagnetic beads coupled with antibodies against the epithelial cell adhesion molecule (EpCAM) are used for CTC enrichment by this method. CTC detection in addition requires presence of epithelial cytokeratins and absence of the leukocyte marker CD45 in nucleated cells. Enumeration of CTCs using the CellSearch method has been approved for clinical use by the Food and Drug Administration (FDA) [28]. A limitation of the CellSearch method is that mesenchymal-type CTCs go undetected as the EpCAM marker becomes down-regulated following epithelial-to-mesenchymal transition (EMT), an important step of tumor cell dissemination [29]. To counteract this limitation, alternative antibody-based CTC capture methods have targeted mesenchymal and stem cell markers [30], but several challenges with this approach remain to be solved, e.g., when mesenchymal markers are expressed on circulating benign cell types [31].

CTC enrichment, however, can be independent of marker expression in the CTCs, either by negative depletion-based strategies or by utilizing differences in physical properties [32]. The exciting prospects of CTCs in cancer management, along with their rarity, has spurred technology development. In particular, advances in nanotechnology, microfluidics, and single-cell sequencing have found applications for CTC enrichment and analyses ex vivo and in vivo [33,34,35,36,37,38,39]. In spite of the plethora of different technological innovations, it remains a great challenge to capture the heterogeneity of CTCs and to preserve their viability and functional characteristics for experimental analyses [37].

## 3. Clinical Relevance of Liquid Biopsies in Solid Cancers

### 3.1. Early Detection of Disease

Early detection of cancer is pivotal for the treatment outcome of most cancers. However, the task is notoriously challenging [40]. Despite this, a few recent, seminal publications have provided evidence that liquid biopsies can become a tool for successful cancer screening [41,42]. The CancerSEEK assay, which is based on detection of both ctDNA and protein biomarkers in blood samples, was tested on 1005 symptomatic patients with eight different solid non-metastatic cancers and 812 healthy control persons [41]. The overall sensitivity at optimal biomarker thresholds was 62% and the specificity 99%. However, the sensitivity for cancer types diagnosed at a late stage (98% for ovarian cancer) was much higher than for cancer types predominantly diagnosed at earlier stages (33% for breast cancer). Caution has also been raised regarding the low false-positive rate reported (1%), as the controls in this study were healthy individuals, whereas a real screening setting would also include persons with inflammatory and other diseases, potentially increasing this rate. Recently, an early version of the CancerSEEK method combined with diagnostic PET-CT was successfully used to identify new cancer cases among 9911 previously cancer-free women [43]. Of the 143 with positive blood testing, 127 (95%) were subjected to PET-CT imaging and 64 (50%) obtained images indicating potential malignancies. Twenty-five of them were subsequently shown to have cancer through biopsy. A new version of the CancerSEEK assay is currently being tested in a multicenter, prospective, observational study aiming to include 1000 subjects with known or suspected cancer in addition to 2000 subjects without known cancer (ClinicalTrials.gov Identifier: NCT04213326) to validate the updated classification algorithm [44]. 

The Circulating Cell-free Genome Atlas (CCGA) consortium recently reported data from an even larger prospective trial, comprising 2484 patients with 50 different cancers and 4207 individuals without cancer [42]. Using targeted bisulfite sequencing to detect ctDNA, they obtained overall sensitivities ranging from 18% in all stage I cancers to 93% in stage IV cancers and a false-positive rate around 1%. For some cancers, such as pancreatic cancer, the sensitivities were considerably higher, yielding hope for a future clinical utility of this technology. Interestingly, a similar approach is currently being investigated in several large ongoing studies (e.g., STRIVE, PATHFINDER, SUMMIT) [45]. Other groups have also demonstrated promising cancer detection using methylation sequencing [12,46]. A research group demonstrated good distinction between 199 cancer patients and 62 healthy control persons in their validation cohort, reporting areas under receiver operating characteristic (ROC) curves from 0.91 to 0.98 [12]. More recently, the PanSeer ctDNA methylation blood test was reported to correctly classify 95% of 98 cancer patients and 96% of 207 samples from healthy control persons [46].

### 3.2. Prognostic Stratification

The evidence for the prognostic value of ctDNA in solid cancers is extensive and has been demonstrated for several different cancers, including breast [47,48], colorectal [49,50], lung [51,52,53], melanoma [14,54], and pancreatic cancer [13,55]. Several studies have indicated an inverse relation between ctDNA level and survival, with increasing levels of ctDNA being significantly associated with poor survival [7,47]. Further, early dynamic changes in ctDNA levels have also been shown to predict survival [52,53]. The evidence of a prognostic value of ctDNA is strongest for patients with inoperable disease [13,49,50], but there is also some evidence for resected patients [13,48]. A major challenge in using ctDNA to predict prognosis in a clinical setting is, however, the lack of standardized cutoff values for ctDNA positivity.

The prognostic relevance of CTCs has primarily been investigated using the CellSearch system, which has FDA approval for prognostication of metastatic breast, colon and prostate cancer. This application has been extended to additional solid cancer types, such as lung and urogenital cancers and malignant melanoma. Associations between ≥5 CTCs and inferior progression-free and overall survival have been demonstrated especially in the metastatic setting [5,56,57,58,59]. However, there is also evidence for prognostic relevance of CTCs in non-metastatic cancer, usually by applying a lower cutoff for CTC positivity (≥1 CTC per 7.5 mL blood) [60,61,62]. Prognostic relevance has also been shown for morphological features of CTCs, such as the association between CTC clusters in the circulation and adverse prognosis [63,64,65,66].

EMT and the reverse process mesenchymal-to-epithelial transition (MET) have been postulated as critical programs for efficient metastasis [29]. A link between CTCs with mesenchymal features and prognosis has been indicated in several studies [31,67,68,69]. However, the evidence for a prognostic value of mesenchymal-like CTCs is currently not as extensive as for the CellSearch system, and further research is required to clarify the role of CTC phenotypes [5,70]. 

### 3.3. Choosing Therapy Based on Liquid Biopsies

The ultimate utility of liquid biopsies in cancer is to guide clinical decision-making. Current evidence supports a predictive value of both CTCs and ctDNA with regard to various cancer treatments (recently thoroughly reviewed in [5,70]), but the data are most convincing for targeted therapies. The best established example is the role of ctDNA Epidermal Growth Factor Receptor (*EGFR*) mutations in the treatment of advanced non-small-cell lung cancer (NSCLC), as emphasized by the FDA approval of the cobas^®^ EGFR Mutation Test v2 (Roche Diagnostics, Indianapolis, IN, USA) and the FoundationOne Liquid CDx tests (Foundation Medicine, Inc., Cambridge, MA, USA). Activating mutations in the *EGFR* gene, either in the primary tumor or in ctDNA, predicts treatment effect of EGFR tyrosine kinase inhibitors, whereas the *EGFR* T790M mutation commonly causes resistance [71,72,73,74]. Moreover, resistance-causing mutations (in the *KRAS, ESR1*, or *PIK3CA* genes) in relation to other targeted treatments have been detected in ctDNA from several cancer types, suggesting a future clinical utility of ctDNA in multiple treatment settings [75,76,77,78]. 

Molecular analysis of CTCs has proven to be predictive in relation to targeted treatment. For example, detection of nuclear-localized androgen receptor (AR) splice variant 7 protein (AR-V7) in CTCs (FDA-approved test) from patients with metastatic castration-resistant prostate cancer predicted a survival benefit if they were treated with taxanes rather than AR directed therapy [79,80]. Moreover, detection of AR-V7 or the neuroendocrine marker synaptophysin on CTCs both suggest resistance to androgen deprivation and AR inhibitor treatments and appears as useful to guide therapy of advanced prostate cancer [81,82]. Other promising cases include ER-expression on CTCs in ER-directed treatment of breast cancer and ALK rearrangements in CTCs from NSCLC patients treated with ALK inhibitors [83,84]. Moreover, methods to culture CTCs and establish CTC-derived explants has opened new possibilities for personalized therapy selection [85].

Furthermore, a survival benefit of radiotherapy in two independent cohorts of operable breast cancer patients was only demonstrated in CTC positive patients [86]. In addition, a survival benefit of using CTC counts to choose between anti-hormone treatment and chemotherapy in metastatic breast cancer has also been suggested [87], whereas copy number aberrations in CTCs from small-cell lung cancer patients were associated with chemo-refractiveness in another study [88].

Immune checkpoint inhibitors have received much attention due to convincing treatment effects for some cancer types [89]. Tumor mutational burden (TMB) in tissue biopsies is an established predictive factor for immunotherapy in some cancers and ctDNA mutations can be used as a surrogate [90,91]. PD-L1 expression on CTCs also seems to have potential for predicting treatment response to checkpoint inhibitors [92].

### 3.4. Disease Monitoring and Early Detection of Relapse

Serial analysis of ctDNA and CTCs has the potential to provide information on disease progression, therapeutic effect and resistance, and tumor evolution. Monitoring of tumor burden for early detection of disease progression and therapeutic effect is currently performed by analysis of conventional protein biomarkers in blood and radiological imaging, methods which suffer from low specificity and/or sensitivity [93]. However, recent studies have demonstrated that an increase in the ctDNA level may reveal disease relapse or progression at the same time or earlier than radiological imaging [48,94,95,96,97]. Moreover, longitudinal monitoring of ctDNA levels is shown to predict treatment efficacy in several cancer types [77,98,99]. Molecular characterization of ctDNA during follow-up may also reveal mutations causing therapy resistance, information that may help guiding treatment decisions in the future [7,47,77].

There are indications that longitudinal changes in CTC enumeration can predict treatment response [59,100], but overall it has been challenging to prove a strong clinical value of CTC monitoring, possibly related to their low numbers [101]. Thus, in the near future we expect ctDNA assessment to be more useful than CTCs for monitoring disease burden, treatment response and early detection of relapse.

## 4. Implementing Liquid Biopsies in Clinical Studies

Experimental evidence for the clinical utility of a liquid biopsy test, and not only its clinical relevance, is a prerequisite for its incorporation in clinical practice [102]. As described above, only a few tests currently have this level of evidence, encouraging the design of new interventional studies where the role of liquid biopsy tests for making treatment choices is evaluated. A large number of ongoing clinical trials now includes liquid biopsies in their trial design (Clinicaltrials.gov). However, only a few of the studies are interventional. Accordingly, the massive focus on prognostic value and monitoring potential in the liquid biopsy field should be shifted towards randomized clinical trials [103]. However, incorporation in such trials depends on promising observational data, emphasizing the cascade of evidence needed for a biomarker to make its way to the clinic [104]. 

### Experiences from a Norwegian Regional Research Program on Liquid Biopsies

We recently finished a consortium-based five-year research program on liquid biopsies entitled “Personalized cancer therapy–biomarkers in clinical trials”, funded by the Western Norway Regional Health Authorities and involving research groups from three Norwegian hospitals. Several observational and interventional research projects were initiated and performed during the project period, providing considerable experience on the implementation of liquid biopsies in clinical studies [14,55,105,106,107,108,109]. The most obvious challenge was the plethora of available techniques for both ctDNA and CTC assessment, and the difficulty to standardize techniques across projects including different cancer types and treatment settings. Standardization was restricted by the continuous development of new technology in the field and a lack of consensus on what to prioritize when choosing technologies [110]. We would also like to emphasize the importance of standardization of pre-analytical conditions like sampling tubes, time from sampling to first centrifugation for ctDNA, storage conditions and times, and transport routines [102]. The requirement for rapid CTC enrichment after sample collection (2–96 h, depending on tube) makes the pre-analytical processing more complicated for CTCs than ctDNA; an argument to prefer ctDNA over CTCs in clinical studies [102,111,112]. During the development of the projects, we also learned that larger sample volumes (>10 mL blood) are preferable to increase analytical sensitivities, especially in non-metastatic settings [113]. 

To ensure successful integration of liquid biopsies in clinical studies, optimal standard operating procedures (SOP) need to be in place before the start of recruitment [110,114]. This will highlight the methodological needs, the choice of sample material, as well as staff needs, for implementing liquid biopsies. It is important to have personnel dedicated for sample handling and processing directly after blood draw, as the half-lives of ctDNA and CTCs are short, and the sample quality will deteriorate rapidly. To ensure efficient analysis of results, it is also important to allocate enough resources for bioinformatics.

## 5. Liquid Biopsies in Clinical Practice 

### 5.1. Liquid Biopsies Currently Applied in Hospital Settings in the Nordic Countries

The implementation of new diagnostic tests in routine analysis in Nordic hospitals is often slower than in other regions because of the strict regulations and reimbursement system used in their public healthcare systems (further discussed in Section 5.2.5). The most established liquid biopsy test in clinical practice is testing for *EGFR* mutations in non-small-cell lung cancer (Table 3). As described above, several sensitizing mutations in this gene are strongly associated with response to EGFR tyrosine kinase inhibitors (reviewed in [115]). Traditionally, tumor tissue has been tested for *EGFR*-mutations, but it is not always possible to collect sufficient tumor samples without placing patients at risk of complications [116]. This is often even more challenging at the time of progression, when a rebiopsy ideally should be collected to individualize subsequent therapy [117]. Fortunately, several studies clearly demonstrate that *EGFR* mutations may be detected in cfDNA for a high proportion of patients with known *EGFR* mutations in their tumor. The response rates to EGFR TKIs are similar for these patients as for patients who are offered this treatment based on the detection of *EGFR* mutations in tissue samples [71,72,73,74,118]. Thus, it is well accepted that peripheral blood tests may replace tissue testing when tumor samples are unavailable or the risk associated with a biopsy is high. A similar approach is also accepted when testing for the most common resistance mutation among patients on EGFR TKIs, the T790M mutation [74]. 

### 5.2. Challenges for Further Integration of Liquid Biopsies in Clinical Practice 

#### 5.2.1. Evidence of Treatment Relevance Needed

**Figure 1:**
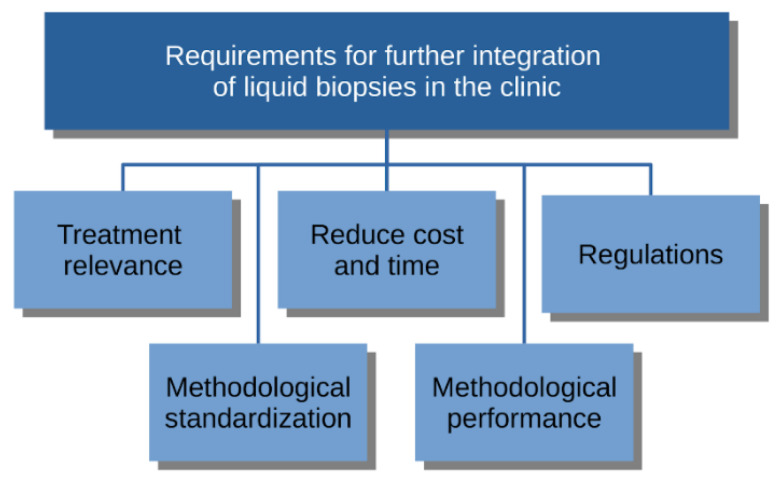
Overview of the most important identified requirements for further integration of liquid biopsies in the clinic.

The multitude of prognostic cancer markers never reaching the clinic emphasizes the main criteria for successful integration in the clinic: relevance for clinical decision-making (Figure 1). We have already described promising research supporting the applicability of liquid biopsies in the choice of therapy and monitoring of treatment effect. Excellent evidence for such utility has been provided for NSCLC and ctDNA, explaining its current role in the clinic. However, for other cancers and treatment settings, there is still a need for more clinical trials [102]. CTC enumerations are not performed routinely in Nordic hospitals, despite the FDA approval, probably because of weak evidence for a predictive value.

The fact that mutations predicting therapy response or resistance can also be used to detect ctDNA emphasizes that targeted biological drugs might be the most likely candidates for development of accompanying liquid biopsy-diagnostics. We anticipate increasing clinical utilization of such ctDNA application, including detection of resistance mutations in relation to EGFR-directed therapy of colorectal cancer and *ESR1* mutations in relation to anti-hormone therapy in breast cancer [75,76]. TMB assessment in relation to immune therapy is another promising candidate for useful application of liquid biopsies in clinical work [90,91]. Recently, several high-impact articles have suggested a role for ctDNA detection in early cancer detection with the possible benefit of treating at a non-metastatic disease stage [11,12,41,43]. Early detection screening programs have been questioned by the risk of overdiagnosis [122] and the establishment of early cancer detection described in these studies is very extensive. However, we believe that the approaches described in these articles can at least be beneficial for high-risk groups. 

#### 5.2.2. Methodological Standardization

The analytical performance of liquid biopsy tests must be assessed in prospective studies using validated protocols to decide clinical validity and utility. So far, most of our knowledge is based on retrospective analysis with poorly described and often quite disparate procedures. Standardization of the complete workflow is pivotal for implementation in routine diagnostics [114]. To address this, the European Liquid Biopsy Society [123] and BloodPAC [124] (USA) have been formed, joining experts from academic and clinical research with diagnostic companies and the pharmaceutical industry. These initiatives seek to establish standardized protocols, mutual validation of methods, data sharing and development of new technology.

The need for standardization includes both the pre-analytical steps, the analysis itself, interpretation of results and reporting. Proper reference materials are useful for both the pre-analytical and analytical validation (further discussed in [125]). The need for improvement, particularly regarding ctDNA, was shown in a recent proof-of-principle study [126]. Important questions are how to report ctDNA levels, and how to determine diagnostic thresholds. Recently, the National Cancer Institute’s Biorepositories and Biospecimen Research Branch has developed cfDNA-specific guidelines in an effort to standardize optimal procedures for sample collection and processing [127].

#### 5.2.3. Cost and Time Requirements

For ctDNA, detection by deep sequencing is far more expensive than techniques based only on PCR, but it is superior for mutation profiling and early detection, as ‘a priori’ mutation knowledge is not required. A major contribution to the cost is the sequencing, which depends on the number of samples sequenced simultaneously, sequencing depth, sequencing reagents and instruments. In sparsely populated areas, such as the Nordic countries, few patient samples can delay turnaround time if sequencing costs are to be kept to a minimum. ctDNA detection by PCR-based methods is faster, cheaper and easier to perform on a small scale, although it is more difficult to generalize for several cancer types. For treatment monitoring, the level of a particular mutation using ddPCR/BEAMing is from our point of view the only affordable approach with sufficient sensitivity at the moment, even if it is limited to patient-specific mutations. However, this may change as sequencing costs are further reduced. Sample collection and processing for CTC analyses, as well as sample shipment in many hospital settings, is also associated with considerable cost and time requirements.

#### 5.2.4. Liquid Biopsy Performance Compared to Routine Diagnostics

Massive evidence for EGFR mutation testing in ctDNA from NSCLC patients demonstrates good concordance with primary tumor assessment and clinical utility of ctDNA in relation to targeted treatment [71,72,73,74]. ctDNA is also more likely to reflect tumor heterogeneity and resistance mechanisms, particularly when multiple metastases are present [76,128]. Conversely, if targeted treatment is chosen based on a minor tumor clone found in ctDNA, a treatment effect on the bulk of the tumor (not harboring the clonal mutation) cannot be expected [102]. Based on published research and clinical experience with genetic diagnostic testing of aggressive acute myeloid leukemia, we foresee a scenario for solid cancers, where multiple mutations represent the oligoclonal repertoire of the tumor [129,130]. Treatment guided by response evaluation through ctDNA can then be tailored to remove the multiple clones by targeted therapy and chemotherapy. 

Another important clinical setting in which liquid biopsies have the potential to replace or reduce routine diagnostics is in the monitoring of tumor development during treatment [6]. Repeated blood sampling is feasible and many of the liquid biopsy-based analyses are both more sensitive and less resource-demanding than radiological imaging [97,131,132]. Nevertheless, this approach is insufficient for a subgroup of patients in most published studies, either because some patients lack liquid biopsy markers or because the increase in marker levels detected by liquid biopsy is delayed compared to radiological evidence of recurrence. By using personalized ctDNA and CTC markers, more sensitive assays, and larger volumes of blood, the detection rates can hopefully be optimized [96,97]. However, it is difficult to imagine a total replacement of radiological imaging in the surveillance of patients with solid cancers. We believe a combination of surveillance methods might be more likely, preferably with more frequent ctDNA assessments than radiological examinations.

#### 5.2.5. Regulations

In Norway, the healthcare system is publically funded and new expensive methods need to be approved by a national health technology assessment before implementation in routine diagnostics. The main topic considered in these processes is the clinical utility in regard to sample type, cancer type, risk of complications, clinical application, and related treatment options. In the evaluation of liquid biopsy methods for predictive purposes both the stakeholders’ perspectives and the availability of targeted drugs can affect the interpretation of evidence and the conclusions [133]. With the multitude of newly suggested liquid biopsy-based approaches, a national health technology assessment is undoubtedly needed, but the guidelines to act upon need to be carefully defined.

## 6. Conclusions and Future Perspectives

Liquid biopsies are currently mostly research tools in clinical trials and in the development of novel diagnostics. The first disease that may take advantage of ctDNA analysis is NSCLC, where it already guides targeted treatment. Recent evidence suggests further application of liquid biopsies in several solid cancers, but validation is required for regulatory approval and establishment of clinical use, not the least to justify the high cost of advanced sample processing and accompanying bioinformatic analysis. Randomized clinical trials using liquid biopsies for treatment decisions are therefore required for further integration of the methods into the clinic. ctDNA seems to have the best potential for becoming a widespread marker in routine diagnostics, whereas CTCs are challenging due to their difficult detection and sampling issues, but may answer more basic questions connected to cancer biology and metastasis.

The required expenses represent a limitation to provide liquid biopsy as a robust tool globally. Clearly, technological progress in microfluidics, computing and electronics will reduce the price level of the diagnostic technology. We need an open discussion about open licenses to secure the benefit of knowledge in all regions. The expected revolution in diagnostics should encourage a focus on this topic for international organizations like the World Health Organization in their effort against cancer.

The full potential for liquid biopsies seems to be in concert with other diagnostic modalities [41]. Imaging diagnostics in cancer is also rapidly developing, and provides anatomical and various functional information about the tumor. Liquid biopsy and functional imaging may be the diagnostics tool combination needed to accelerate precision medicine in cancer.

## Figures and Tables

**Table 1:** ctDNA markers and detection methods.

Marker	Detection Chemistry	Advantages	Disadvantages
Point mutations	PCR/digital PCR	High sensitivity, quick, inexpensive	Few targets per analysis, only pre-identified mutations can be analyzed
Point mutations	Deep sequencing	High-throughput, detection of previously unknown mutations, tumor mutation burden (TMB) assessment	Moderate sensitivity, expensive, time-consuming
Copy number variations/trans-locations	PCR/digital PCR	High sensitivity, quick, inexpensive	Custom-designed assays needed, only pre-identified aberrations
Copy number variations/trans-locations	Low-pass whole-genome sequencing, targeted sequencing	Many and unknown targets	Low sensitivity, expensive, time-consuming
Hyper-methylation	Methylation-specific PCR	High sensitivity, low cost	Few targets per analysis, only pre-identified targets
Hyper-methylation	Bisulfite-sequencing	High-throughput, many targets	Low sensitivity for single markers, expensive

**Table 2:** Circulating tumor cell (CTC) enrichment methods.

CTC Feature	Enrichment Method	Advantages	Disadvantages
Size	Size filtration	Simple, inexpensive, marker-independent	Low purity, loss of small CTCs
Size and deformability	Microfluidics	Easily automatable, marker-independent	Varying purity, loss of small CTCs, time-consuming
Density	Density gradient centrifugation	Simple, inexpensive, marker-independent	Low purity, potential loss of CTC clusters
Electrical charge	Dielectrophoresis	Marker-independent	Low CTC recovery, low purity, low throughput
Surface markers	Positive immunomagnetic selection	High recovery, high purity.	Marker dependent
Absence of surface markers	Immunomagnetic depletion	Marker-independent, high recovery, high purity	Loss of CTCs adhering to white blood cells

**Table 3:** Liquid biopsy applications currently in use or near use in Nordic clinical practice.

Clinical Status	Liquid Biopsy Test	Method	Clinical Application	References
Currently in use	cfDNA *EGFR* gene mutation testing	Quantitative PCR	Predictive for EGFR-directed treatment (TKI inhibitors) of advanced lung cancer	[71,72,73,74,115,117,118]
Food and Drug Ad-ministration (FDA) approved	FoundationOne Liquid CDx multigene panel (incl. *EGFR, ALK, PIK3CA, BRCA* genes)	Targeted sequencing	Predictive for targeted treatment of metastatic lung, prostate and breast cancer	[119,120,121]
FDA approved	cfDNA *KRAS* and *NRAS* gene mutation testing	BEAMing, Digital PCR	Predictive for EGFR-directed treatment of metastatic colorectal cancer	[75]
FDA approved	cfDNA TMB testing	Targeted sequencing	Predictive for treatment of several solid cancers with immune checkpoint inhibitors	[90,91]
FDA approved	AR-V7 splice variant testing in CTCs	Immunofluorescence	Treatment choices for metastatic castration-resistant prostate cancer	[79,80]
FDA approved	CellSearch CTC enumeration	Immuno- logical enrichment/ staining	Prognostic stratification of breast, prostate and colorectal cancer	[58,60]

## Data Availability

Data sharing not applicable. No new data were created or analyzed in this study.
